# Barriers and Facilitators to Promoting Resilience to HIV/AIDS: A Qualitative Study on the Lived Experiences of HIV-Positive, Racial and Ethnic Minority, Middle-Aged and Older Men Who Have Sex with Men from Ontario, Canada

**DOI:** 10.3390/ijerph18158084

**Published:** 2021-07-30

**Authors:** Renato M. Liboro, Sherry Bell, Brandon Ranuschio, Lianne Barnes, Jenna Despres, Aruna Sedere, Trinity Puno, Paul A. Shuper

**Affiliations:** 1 Department of Psychology, University of Nevada, Las Vegas, NV 89154, USA; bells12@unlv.nevada.edu (S.B.); fraga@unlv.nevada.edu (B.R.); lianne.barnes@unlv.edu (L.B.); despres@unlv.nevada.edu (J.D.); sedere@unlv.nevada.edu (A.S.); puno@unlv.nevada.edu (T.P.); 2 Institute for Mental Health Policy Research, Centre for Addiction and Mental Health, Toronto, ON M5S 2S1, Canada; paul.shuper@camh.ca; 3 Dalla Lana School of Public Health, University of Toronto, Toronto, ON M5T 3M7, Canada

**Keywords:** barriers and facilitators, resilience to HIV/AIDS, racial and ethnic minority, middle-aged and older, men who have sex with men

## Abstract

Evidence-based research has highlighted the need for exploring factors that support the mental health of men who have sex with men living with HIV/AIDS (MSMLWH), and environmental influences that promote their resilience to HIV/AIDS. This exploratory study utilized a community-based participatory research approach to investigate barriers and facilitators to promoting resilience to HIV/AIDS, specifically among racial and ethnic minority, middle-aged and older MSMLWH, a population that continues to be significantly impacted by HIV/AIDS today. This collaborative, qualitative study recruited participants who identified as racial or ethnic minority MSMLWH, were aged 40 or older, and resided in Ontario, Canada. Participants (*n* = 24) discussed in their interviews barriers and facilitators to promoting resilience to HIV/AIDS, which they recognized from their lived experiences. Utilizing thematic analysis, themes related to barriers and facilitators to promoting resilience to HIV/AIDS were identified. Themes related to identified barriers included: (1) language proficiency, (2) racism, (3) pernicious norms in North American gay culture, and (4) HIV stigma. Themes related to identified facilitators included: (1) compartmentalization, (2) perseverance, and (3) community-based health and social services. This article discusses the implications of the study’s findings, particularly on how they may influence the development of future services for racial and ethnic minority, middle-aged and older MSMLWH.

## 1. Introduction

It is a critical time to explore the resilience of people living with HIV/AIDS (PLWH). In light of the global spread of the novel coronavirus (i.e., SARS-CoV-2) that began in 2020, the World Health Organization (WHO), the Center for Disease Control and Prevention (CDC), and numerous researchers have highlighted the increased risk for severe physical and mental health outcomes and death due to the coronavirus disease 2019 (i.e., COVID-19) among PLWH compared to the general population [1,2,3]. This increased risk for severe physical and mental health outcomes and death due to COVID-19 has been particularly salient for PLWH in North America who are racial and ethnic minorities [1,2,4,5], older adults [1,3], and gay, bisexual, or of other men who have sex with men (MSM) [2,5,6,7]. Although recent literature has discussed the likelihood of increased risk of loneliness among PLWH due to pandemic-related social distancing measures, as much as 87% of PLWH have already reported experiences of loneliness prior to the COVID-19 pandemic [8]. The negative disruptions to HIV health services that have been brought about by the COVID-19 pandemic have not only prompted governments to organize solutions, such as community-based initiatives engaging PLWH, but have also shone a spotlight on the adverse risks of isolation that PLWH have experienced even before the COVID-19 pandemic emerged [9]. Such biopsychosocial and structural risks have underscored how critical it has been to explore the resilience of racial and ethnic minority, middle-aged and older MSM living with HIV/AIDS (MSMLWH), as well as the factors that serve as barriers and facilitators to social support and resilience-building in lesbian, gay, bisexual, trans, and queer (LGBTQ) communities, healthcare facilities, and relevant community-based organizations. 

PLWH have historically experienced health complications related to social isolation due to HIV stigma, even more so for PLWH who have come from traditionally underserved populations [10,11,12,13,14,15]. Health complications related to such social isolation have included hypertension, higher rates of depression and depressive symptoms, lower levels of physical activity, and higher levels of anxiety [12,16,17,18]. As much as prior studies have determined a distinct association between stigma impacting racial and ethnic minorities living with HIV/AIDS and negative mental health outcomes across Black, Latiné, and Aboriginal populations, research has also documented coping mechanisms that racially and ethnically diverse individuals have developed to promote resilience in response to an HIV/AIDS diagnosis [19]. Extant research on the resilience of PLWH has primarily focused on experiences at the individual level of resilience. Consequently, existing literature has prompted researchers to examine resilience at structural and social levels [20]. This study, which was initiated a little over a year prior to the start of the COVID-19 pandemic (i.e., February 2019), utilized a community-based participatory research (CBPR) approach for the express aim of identifying and investigating barriers and facilitators to promoting resilience to HIV/AIDS based on the perspectives and lived experiences of racial and ethnic minority, middle-aged and older MSMLWH. 

### 1.1. Resilience

When operationalizing resilience in HIV/AIDS populations, academic literature has typically defined resilience as the ability to acclimate psychologically, behaviorally, and/or socially in light of adversities, through the support of the systems within an individual’s socioecological model (i.e., individual, family, and community) [21,22,23]. For sources of support, the focus has primarily been at the individual level, looking at the role of individual attributes such as high levels of optimism, acceptance, will to live, self-esteem, fighting spirit, and conscientiousness [24,25,26,27]. Dulin and colleagues [22] found a paucity of research on the roles of community-based resources and social systems in promoting resilience to HIV/AIDS. In fact, their review had been able to cite only one study that observed the role of community centers and their positive influence on PLWH [28].

To illustrate the value of community-based resources in addition to individual traits, it would be informative to look at optimism, a key factor in resilience-building. Individuals living with HIV/AIDS have consistently reported their lived experience of challenge, as well as their lived experiences of resilience. Lived experiences of resilience to HIV/AIDS have involved sustaining optimism in their pursuit of future goals and maintaining good health in the coming years [24,29,30]. Further analysis of this role of optimism in resilience has revealed a crucial need for individuals to gain more knowledge about HIV/AIDS, which could be improved and supported through community discussions, and provided by healthcare facilities that do not stigmatize PLWH [31]. These findings have echoed the WHO’s call for the development of healthcare facilities that are community-based, and the need for resources beyond the individual level to consider programs tailored to appropriate populations that have been noted in prior research [32]. For instance, a community-driven intervention for HIV prevention for young males has been successfully developed and implemented by other young males, thus finding effective results for reducing risk [33]. While programs to prevent HIV/AIDS have been explored in community-based approaches, literature that explores different sources of resilience for those living with HIV/AIDS beyond the individual level, and the role of community services and healthcare facilities to promote resilience to HIV/AIDS has been limited [33,34,35,36,37].

### 1.2. The Role of Community-Based Health and Social Services 

While the perspectives of racial and ethnic minority, middle-aged and older MSMLWH have been typically underrepresented in research, some work has been done to explore the characteristics of support and healthcare provided for this population [38,39]. Specifically, the quality of both emotional and instrumental social support from healthcare providers for PLWH and AIDS service organizations have been related to an increased engagement in HIV treatment [40,41,42]. While qualitative reports have revealed barriers to HIV testing (i.e., stigma, discrimination, and confidentiality) and possible structural changes aimed at encouraging MSM to seek HIV treatment (i.e., peer social support; affordable, specialized care), the literature has also called for increased investigations on access to care, and the level of trust involved between organizations, providers, and patients [42,43]. In fact, the need to investigate resources for older MSM has been pointed out further from evidence that specifically notes the high value that patients have for trust amongst clinicians and service providers [43], encouraging researchers to gather more data looking into the levels of trustworthiness of healthcare and service organizations. In this exploratory study, we employed a CBPR approach to further investigate the care, trust, and access to resources available to racial and ethnic minority, middle-aged and older MSMLWH; a specific subpopulation that has experienced compounding life challenges associated with their distinct, but often marginalized identities since the start of the HIV/AIDS epidemic. A description and discussion of the findings of factors that influence and promote resilience of HIV-positive, racial and ethnic minority, middle-aged and older MSM to HIV/AIDS will be presented. In particular, perspectives of racial and ethnic minority, middle-aged and older MSMLWH will describe barriers and facilitators to promoting resilience to HIV/AIDS based on their lived experiences. 

## 2. Materials and Methods

### 2.1. Participants

The findings discussed in this article are part of a larger study that was designed to examine factors that help promote resilience to HIV/AIDS based on the perspectives of relevant community stakeholders. The larger study recruited 55 middle-aged and older MSM from across Central and Southwestern Ontario, Canada, all of whom participated in semi-structured interviews. Participants were recruited with the support of LGBTQ agencies and AIDS service organizations (ASOs). The inclusion criteria consisted of: (1) age of 40 years or older, (2) willingness to confidentially disclose their HIV status, (3) residence in Ontario, Canada, and (4) self-identification as a man who has had sex with another man at least once in the past year prior to study participation. Among the 55 participants, the slight majority (51%, *n* = 28) identified as belonging to racial or ethnic minorities. Specifically, participants identified as African Caribbean Black (16%, *n* = 9), South/Southeast/East Asian (15%, *n* = 8), Hispanic/Latino (9%, *n* = 5), West Asian/Middle Eastern (5.5%, *n* = 3), and Aboriginal/Indigenous (5.5%, *n* = 3). Among the racial or ethnic minority participants, the majority were HIV-positive (86%, *n* = 24). The ages of the racial and ethnic minority participants ranged from 40 to 77, with the mean age of 54, and a majority coming from the 50–54 age group (24%). While several themes were identified from the entire interview data of the larger study during the analytic process, this article focused specifically on themes pertaining to the perspectives and lived experiences of HIV-positive, racial and ethnic minority, middle-aged and older MSM (44%, *n* = 24). This study was approved by the Research Ethics Board of the Centre for Addiction and Mental Health, Toronto, Ontario, Canada. Participants were assigned pseudonyms from the time of their interview, and those pseudonyms were used to represent them in this article. Participants each received a $25 CAD (~$20 USD) for their participation.

### 2.2. Materials

Prior to their semi-structured interviews, the 24 participants were asked for sociodemographic information related to their race/ethnicity, age, how they chose to identify themselves, and the geographical location of their residence (see Table 1). An interview guide with five sections and 18 open-ended questions that were formulated with the input and feedback of the study’s community advisory board (CAB) was used to inquire about the participants’ individual experiences and factors that helped promote their resilience to HIV/AIDS, including experiences surrounding community-based services that may have contributed to promoting their resilience (see examples of questions per section in Table 2). For each experience, participants were asked to elaborate on their access to services, and the challenges they encountered while availing themselves of these services in the community. Additional interview questions explored personal health practices and coping strategies related to promoting their resilience. 

### 2.3. Procedure and Data Analysis

Interviews were transcribed verbatim and coded inductively by a panel of three coders with relevant lived or work experiences engaging with HIV-positive, racial and ethnic minority, middle-aged and older MSM. In collaboration with community partners, coders reviewed transcribed interviews and utilized thematic analysis [44] to analyze the qualitative data. The panel of coders, consisting of the first author and two research assistants who performed the interviews, conducted the initial step for the thematic analysis of the interview data. As separate coders, the first author and research assistants reviewed and coded eight representative (in terms of race/ethnicity, age, etc.) transcripts for major themes, sub-themes, and representative codes and quotes. The separate coders met to compare themes, sub-themes, codes, and quotes in order to finalize a thematic codebook, which the first author utilized to code and analyze the remaining 16 transcripts for the creation of a comprehensive, community report with deidentified and aggregated data. The rest of the research team and the study’s CAB subsequently reviewed and provided their input on the community report. The community report was later modified to incorporate the input provided by the research team and the study’s CAB, and then shared with community partners for dissemination and knowledge transfer and exchange. This process was conducted using different techniques to establish confirmability (i.e., reflexivity, analyst triangulation), credibility (i.e., source triangulation), dependability (i.e., external audit), and transferability (i.e., thick description), as well as uphold the trustworthiness and rigor of the study’s findings and analysis [45].

## 3. Results

During the qualitative interviews, participants shared their perspectives on factors that posed as barriers and facilitators to promoting their mental health and resilience to HIV/AIDS based on their lived experiences as racial and ethnic minority, middle-aged and older MSMLWH. Thematic analysis of their interview data identified several themes related to barriers and facilitators to promoting resilience to HIV/AIDS. Themes related to identified barriers included: (1) language proficiency, (2) racism, (3) pernicious norms in North American gay culture, and (4) HIV stigma. Themes related to identified facilitators included: (1) compartmentalization, (2) perseverance, and (3) community-based health and social services. These themes are the most prominent topics and issues that were discussed by our participants and are described with specific and illustrative excerpts in the following sections. (see Table 3).

### 3.1. Themes Related to Barriers to Promoting Resilience to HIV/AIDS

Over half of the racial and ethnic minority, middle-aged and older MSMLWH (58%, *n* = 14) who participated in the study migrated to Canada from a different country. Among these participants, slightly over half (57%, *n* = 8) migrated for the express purposes of settling as immigrants and making Canada their new home. Slightly less than half (43%, *n* = 6) of these migrant participants came as refugees or asylum seekers, mostly because they were forced to flee their country of origin due to fear or threat of physical harm or impending danger as persecuted sexual minorities. As migrants, many of these participants moved to Canada as young or middle-aged adults, having lived for most of their lives in predominantly non-Western cultures, and communicating in different languages other than English or French. Based on their experiences, over half of the participants described barriers to promoting HIV resilience as associated with cultural and/or language differences. It was also evident that most of the barriers many of our participants encountered were intrinsically tied to their immigration to Canada. 

#### 3.1.1. Language Proficiency

Among the participants who migrated to Canada as young or middle-aged adults, proficiency in reading, writing, speaking, and understanding the English or French language was perhaps one of the earliest and most frustrating barriers to promoting resilience to HIV/AIDS that they encountered. Whether they were already HIV-positive when they migrated to Canada, or became HIV-positive shortly after their move (most found out about their HIV diagnosis within two years of migrating), the majority of these participants found it extremely difficult to access information, health services, and social support programs for PLWH in the first few years after their move, specifically because of the language barriers. It was an additional struggle to have to learn to communicate in English or French while seeking HIV/AIDS care and services from the community. These participants were aware that there were HIV services and programs available in the community that could meet their needs, but they still needed to overcome the language barriers first, as an initial but significant hurdle. Roberto (41 years old, bisexual), who migrated from Venezuela with a known HIV diagnosis, shared in his interview:

I wanted to ask the immigration doctor where or how I can find help to deal with the HIV, but the language barrier made it so hard for me to understand him and let him know what I needed. I realized that this was a problem many immigrants faced when they tried to access healthcare and services to meet their needs.

#### 3.1.2. Racism

Although language barriers were a common experience among many participants, most reported that having to improve their English or French language proficiency to access support services and promote their resilience was a barrier that they were eventually able to overcome after some time. It was not nearly as persistent and pervasive as racism, a barrier which they continued to encounter even as they aged with HIV/AIDS. As certain issues such as homo/bi/transphobia, which impacted most of the LGBTQ community, bound people of different races and ethnicities together, racism was an issue that continued to pull different members of the LGBTQ community apart. According to the participants, although racism was still very much in the fabric of the larger society, they believed it was even more pronounced in the LGBTQ (especially in the gay) community. In larger society, they would experience subtler (and occasionally overt) forms of racism at work, or while accessing healthcare and social services. According to Amir (46 years old, gay), a Middle Eastern migrant who was diagnosed with HIV shortly after he moved to Canada:

I was fired from work for supposedly smelling differently and making other staff feel uncomfortable.

Gary (61 years old, two-spirit), a First Nations participant, talked about his experiences at a community clinic:

The Aboriginal [sexual health and HIV] services in Downtown were based on stereotypes and have been ghettoized.

However, in the gay community, racism was often displayed more blatantly, and even unapologetically. According to Irving (50 years old, gay), a Southeast Asian participant:

It would say right there on their [dating] profile, ‘no fatties, no femmes, no Asians.’ When you confront them, they would say, ‘It’s not racism, it’s preference.’ But if you read the rest of their profile carefully, it’s racism.

Randy (41 years old, bisexual), an African Caribbean Black (ACB) respondent, echoed this sentiment:

When it comes to dating, the pecking order in this community is based on the choices of [racist] White, young men. Racism in the gay community is more prominent than in the straight community!

Interestingly, participants shared that racial prejudice and bias were not only displayed by White (often Canadian-born) MSM, but it was also explicitly evident among different racial and ethnic communities. As another challenge to deal with in their daily lives, racism (and ethnocentrism) exacerbated the already difficult circumstances that the participants experienced while trying to promote their resilience to HIV/AIDS. Juan (47 years old, gay), a Latino participant from Downtown Toronto, pointed out an observation he made in certain Spanish-speaking communities:

One set of gay Latinos from one country feels superior over another group of gay Latinos from another country. They brought attitudes of superiority from their own country to Canada with them.

#### 3.1.3. Pernicious Norms in North American Gay Culture

Several participants expressed that in North American gay culture, many of the existing norms were pernicious, and that there seemed to be no hope for this culture to change. According to these participants, the norms in North American gay culture, which they believed were especially prevalent in metropolitan cities like Toronto, were pernicious because it was not uncommon for many MSM to ‘play mind games’; be insincere, malicious, or full of attitude; and not call out lies, particularly when it comes to getting to know one another or dating. Moh (54 years old, gay), a Middle Eastern participant who has had HIV/AIDS for over five years, described his experiences:

As a Muslim immigrant, I found the gay culture of ‘playing mind games’ really difficult. A lot of it is cold and superficial.

It seemed that there was also a number of ‘rules’ that everybody had to follow, some of which the participants felt, would place them at increased risk for sexually transmitted infections (STIs) and negative mental health consequences. These were norms that many of them were not comfortable following. Jose (48 years old, gay), another Latino participant, explained:

They would mock you if you don’t do drugs. They would mock you for not having sex without condoms. Unless you follow their ‘rules’ and do drugs during sex without condoms, you don’t get to ‘play’.

Since many of the participants were raised in a different culture, not only did they find it difficult to engage with other MSMLWH, but they also found this difficulty adjusting to these norms a huge barrier to promoting their reslience to HIV/AIDS. 

#### 3.1.4. HIV Stigma

Most of the participants had decades of personal experiences as racial and ethnic minority MSM, first at risk of, and then later, living with HIV/AIDS. Apart from racism, the one other societal barrier to promoting their resilience that participants claimed as having existed for as long as they could remember was HIV stigma. Participants discussed HIV stigma with frustration, and their discussions on it revolved around three major areas. First, they talked about how HIV stigma resulted from ignorance, and how this ignorance subsequently led to rejection by family and friends. Being rejected by family members and close friends was perhaps the most painful consequence of HIV stigma. Malcolm (57 years old, bisexual), an ACB participant from the Greater Toronto Area, recalled how stigma was such a barrier to accessing support services in the late 1990s:

It was sad. People, especially Black men who were too afraid to come out and risk their families knowing that they may have had the virus, got help just way too late.

Second, participants described how, even in recent years, HIV stigma was still prevalent in the healthcare system in the 21st century. Derek (63 years old, gay), an immigrant from China who was diagnosed with HIV a year after he moved to Toronto, described his experience:

A nurse at a clinic said to me, ‘I don’t really deal with [HIV-positive] people like you.’ Then, she just walked away from the room and never came back.

Finally, participants also talked about how stigma perpetuated the criminalization of HIV in society. They vividly recalled when the disclosure of one’s HIV status became a legal issue, and after decades later, recognized that provincial laws have not caught up with the science of HIV/AIDS and modern medicine. Ricky (50 years old, gay), a participant from El Salvador, remembered when he first came to an upsetting realization:

I slowly realized how and why they kept criminalizing HIV all these years. It’s all about the enormous amount of stigma attached to it.

### 3.2. Themes Related to Facilitators to Promoting Resilience to HIV/AIDS

Conversely, participants also shared a number of factors that facilitated the promotion of their resilience to HIV/AIDS over the years. They talked about personal strategies to lessen the amount of additional stressors in their lives so that they could focus on coping with the clinical and social impacts of HIV/AIDS; some individual attributes that they felt they needed to develop or sustain along the way; and the best sources of support they appreciated, which helped them get through their challenges related to HIV/AIDS, and promote their resilience. 

#### 3.2.1. Compartmentalization

Although several participants would be the first to admit that it was not the most ideal strategy to utilize in their efforts to overcome barriers to promoting their resilience, compartmentalization was not an uncommon path that participants took in order to prevent different sources of stress, such as racism and HIV stigma, from complicating their personal journeys to surviving, and eventually, thriving with HIV/AIDS. For some racial and ethnic minority, middle-aged and older MSM who joined the study, compartmentalizing the different aspects of their lives seemed to be the most natural and convenient strategy for dealing with the syndemic factors that contributed to the compounding life challenges that were associated with their distinct, but often marginalized, intersecting identities (i.e., as sexual minorities, racial or ethnic minorities, PLWH, older adults, migrants and newcomers). Some participants found that compartmentalization was a temporary but effective protective factor that allowed them some respite and provided them some time to sort out their various life challenges, which would have likely overwhelmed them completely had they not made the conscious choice to separate the different compartments of their lives at that time. For the most part, participants kept everyone and everything related to their social and sexual activities, and their HIV/AIDS, separated from other people and aspects from their family and/or work lives. Most importantly, participants were able to avoid judgment, humiliation, and even rejection from family, friends, and colleagues, by using compartmentalization as a behavioral strategy. According to participants, despite the possible risk of experiencing fragmentation or isolation while using compartmentalization as a strategy, the ability to avoid judgment, humiliation, and rejection provided benefits to their mental health and wellbeing that undoubtedly outweighed any risks. 

A significant part of their purposeful compartmentalization included selective disclosure—choosing only a select few to disclose specific matters related to their sexual orientation or activities, and/or HIV status, to specific people. This was true for participants from all the racial and ethnic minorities who participated in the study, but most especially among the Southeast Asian participants. Yun (43 years old, gay), one of the Southeast Asian participants from Downtown Toronto, explained:

In our [Chinese] culture, it’s like in the US military when it comes to our sexual orientation, it’s don’t ask, don’t tell. Your family won’t ask, and you shouldn’t tell. I just separated my work life in Chinatown from my sex life in the village. I needed to keep them separate until I could figure things out. For the longest time, it worked for me.

#### 3.2.2. Perseverance

Most of the participants discussed in their interviews the merits of developing and nurturing certain personal attributes, which they found useful in their efforts to promoting their resilience to HIV/AIDS. For example, numerous participants felt they needed to have greater perseverance to survive the clinical and social impacts of HIV/AIDS, especially during the early years after they learned of their diagnosis. Like many other aging MSMLWH in the last 30 to 40 years, the participants expressed that they needed to be optimistic and proactive in terms of researching relevant information on HIV/AIDS, finding competent healthcare providers, obtaining appropriate medications and services, and staying financially stable to survive. However, as aging racial and ethnic minority MSMLWH, they felt they needed to persevere even more than their White counterparts because they also had to deal with issues brought about by language barriers, racism, and having to adjust to pernicious norms in North American gay culture, in addition to the issues brought about by the clinical effects of HIV/AIDS and HIV stigma. Edgar (50 years old, gay), an ACB participant who was diagnosed with HIV prior to moving to Canada, commented:

It’s rough enough that I had to deal with HIV stigma in the [gay] community…I needed to persevere against all odds because I had to regularly deal with racism too!

#### 3.2.3. Community-Based Health and Social Services

Apart from having discussed the merits of compartmentalization as a provisional strategy, and individual attributes such as perseverance to promote their resilience, participants emphasized the considerable value of having community-based health and social services that often helped offset their lack of inherent privilege or personal resources to access the care and support they needed. In particular, participants talked about the availability of community health centers in Downtown Toronto and ASOs in their respective neighborhoods or regions. 

Several participants reported that there were times in their life that they endured prolonged periods of financial instability, food insecurity, and homelessness. These were times when they were also unable to find or avail of family physicians, HIV specialists, psychiatrists, counselors, or case workers who could provide them with the continuity of care they needed to manage their HIV/AIDS. In order to access the care they needed, participants relied heavily on the presence of community health centers in lower income neighborhoods and underserved areas of the city. These community health centers provided them access to providers who looked after the management of their HIV/AIDS and other basic health and service needs. Although they may not have had the same healthcare provider each time they went for a clinic visit, the community health centers were places that kept their medical records and referral documents on file, which helped facilitate the continuity of their HIV care. Kwaku (60 years old, gay), an ACB participant who moved to Toronto almost a decade ago, recounted an experience he had that made him so grateful for community health centers:

I was afraid to go. Eventually, I had to. I explained to the doctor at the community health center that I was almost out of meds. I hadn’t been out of medications since I started taking them, so I was really scared. The doctor did not even ask me if I was documented. I was so relieved! He took me in as his patient, and got me my meds on time.

Almost all of the participants had the opportunity to avail themselves of the services and programs of ASOs, either in downtown Toronto or within their region, if they resided in the Greater Toronto Area or elsewhere in Southwestern Ontario. More than half of the participants availed of the services and programs of ASOs on a continuing basis, and many of them utilized the services and programs of at least two ASOs at the same time. The participants revealed that there were three types of ASOs they could access, especially in downtown Toronto. The first type would be the mainstream ASOs. These would be the largest, and likely, the longest-running ASOs in the major cities that had the highest number and widest array of services and programs for their clients. These mainstream ASOs also had the most financial resources, which they gained either from government funding, grants, donations, or fundraising activities. The mainstream ASOs would primarily focus on providing health-related services, mental health care, counseling, education-based services, social support programs, and practical aid. The second type of ASOs would be the specialized ones. These specialized ASOs would be smaller in size in terms of their number of staff members, and often, the premises of their offices. They would be specialized because they would offer programs that would focus specially on certain services only, such as information exchange, counseling, hospice care, day health and wellbeing, housing security, legal and immigration services, or the distinct needs of PLWH who have been incarcerated. Finally, the third type of ASOs would be the ethnoracial minority-serving ASOs. These would be the ASOs that catered services to PLWH of a specific race or ethnicity. Examples of these would be ASOs that initiated and sustained programs specifically for Aboriginal/Indigenous, African Caribbean Black, Asian, or Latiné PLWH. 

Although some participants on occasion used services from the specialized ASOs, many participants admitted that they mostly utilized a combination of the services and programs offered by the mainstream ASOs and the smaller ethnoracial minority-serving ASOs. According to the participants, the mainstream ASOs offered many of the relevant and practical programs they needed to promote their resilience to HIV/AIDS. The mainstream ASOs would likely have greater continuity of services, and a greater number of clients and volunteers. This meant that they would be able to consistently avail themselves of services and programs in these mainstream ASOs for longer periods of time, and would have opportunities to meet and actively engage with more MSMLWH of different races and ethnicities. On the other hand, the ethnoracial minority-serving ASOs offered services and programs that took into serious consideration their needs related to their own cultures, traditions, and languages. In these ASOs, participants were able to more easily identify and feel more comfortable with service providers who were of their same race or ethnicity, and spoke their own language. Juan (47 years old, gay) voiced his appreciation for one of the Latiné-serving ASOs in Downtown Toronto:

Although it doesn’t have as much resources as the bigger ASOs, [name of Latiné-serving organization] is really good for newcomers with language barriers and don’t speak English much…it helps them with translation services and eventually improving their English.

Even more importantly, it was in the ethnoracial minority-serving ASOs where participants were able to experience a greater sense of belonging, acceptance, understanding, and even sense of family. Jin (49 years old, gay), who concurrently went to one of the larger, mainstream ASOs and one of the Asian-serving ASOs in the past few years, explained why he enjoyed accessing services at an organization that was explicitly ethnoracial minority-serving:

It’s great to go to [name of Asian-serving organization]! I feel more at home there. They have case workers who look like me, and have the same or a similar culture and language as mine.

Participants revealed that they were often able to avoid and overcome more barriers to promoting resilience to HIV/AIDS (i.e., language proficiency, racism, and HIV stigma) by accessing services and programs at ethnoracial minority-serving ASOs. This revelation underscored the critical importance of having ethnoracial minority-serving ASOs available in communities with large populations of racial and ethnic minority, middle-aged and older MSMLWH. 

## 4. Discussion

Inherent to possessing multiple identity domains that have placed them at increased risk of experiencing health and psychosocial challenges, racial and ethnic minority, middle-aged and older MSMLWH have consequently encountered barriers to promoting their resilience to the clinical and social impacts of HIV/AIDS since the start of the epidemic. According to the perspectives and lived experiences of our participants, the most prominent barriers they have encountered to promoting their HIV resilience over the years include factors such as *language proficiency*, *racism*, *pernicious norms in North American gay culture*, and *HIV stigma*. 

Research has shown that a lack of *language proficiency*, particularly among migrants and newcomers, not only exacerbates the vulnerability of racial and ethnic minority MSM to HIV/AIDS [46], but also limits their access to appropriate screening and testing, timely care, and relevant prevention and treatment services [46,47,48,49]. These types of access restrictions due to language barriers have been found to lead to poorer patient assessment, misdiagnosis, delayed care, incomplete understanding of prescribed treatment, and impaired confidence in health services [47], which could understandably have resulted in negative health outcomes among racial and ethnic minority MSMLWH. 

Compared to language barriers, *racism*, along with *ethnocentrism*, have been found to affect racial and ethnic minority MSMLWH in more profound ways. In addition to affecting their sexual health in similar ways that language barriers have [50], research has documented that racism and ethnocentrism have adversely affected health reporting on the HIV care and treatment of racial and ethnic minority MSMLWH [51], their perceptions of HIV risk and personal beliefs on HIV/AIDS [52,53], and the impact of HIV prevention strategies that they have successfully adopted [54]. 

Researchers have been fastidious in pointing out the importance of considering not only racial, ethnic, and cultural factors in the development of HIV interventions for racial and ethnic minority MSMLWH, but also the influence of the *dominant gay culture* on the success of general HIV programs for MSMLWH of color [55]. The prevailing norms of the dominant gay culture they are immersed in have historically been found to mix, and many times, clash, with the traditional cultural norms of both migrant and native racial and ethnic minority MSM; thus, presenting them with evolving challenges related to addressing pertinent concerns about their care [56]. Researchers have also particularly emphasized that dominant gay culture is by no means unchanging, and that its dynamically changing norms are difficult to navigate since they consistently evoke new sexual interactions among racial and ethnic minority MSMLWH [57]. 

Very much like racism, ethnocentrism, and some of the pernicious norms of North American gay culture that were described in this article, *HIV stigma* has been a pervasive factor that has proven to be a significant barrier to managing sexual health risks behavior and accessing needed HIV/AIDS care [58]. Even though HIV stigma has insidiously affected not only racial and ethnic minority but all MSMLWH, it has actively interacted with other stigmas rooted from other marginalized identities that have disproportionately impacted racial and ethnic minority MSM since the 1980s [13,50]. The persistence and negative impacts of HIV stigma on racial and ethnic minority MSM in healthcare settings has been clearly documented in empirical studies [59,60], and has drawn much attention to the need for frameworks, programs, and interventions that would directly address the interdependence of such co-occurring multiple stigmas [61,62,63].

Based on the lived experiences of our participants, factors such as *language proficiency*, *racism*, *pernicious norms in North American gay culture*, and *HIV stigma*, have not only been considerable obstacles to accessing appropriate HIV screening, testing, prevention and treatment services, and vital care, but they have also been significant barriers to promoting their resilience to the clinical and social impacts of HIV/AIDS. Interestingly, although *ageism*, *homo/biphobia*, and *xenophobia* were mentioned as barriers by some participants, there was not enough emphasis or discussion on them in the study interviews to warrant their inclusion as superordinate themes or as crucial findings of our study. Reasonable explanations to this lack of emphasis on ageism, homo/biphobia, and xenophobia include the possibility that the other barriers repeatedly discussed were so prominent that they overshadowed the participants’ lived experiences related to ageism, homo/biphobia, and xenophobia; or perhaps, many of the participants did not actually recognize such lived experiences as distinctly as the themes that we identified from the interviews as their barriers. Conversely, our participants’ lived experiences have also revealed that factors such as *compartmentalization*, *perseverance*, and *community-based health and social services* have been important facilitators to promoting their HIV resilience and essential to helping them overcome obstinate barriers. 

Research has examined and found a significantly favorable and positive association between supportive social and structural contexts and HIV transmission reduction behaviors of racial and ethnic minority MSMLWH [64,65]. This finding has provided the foundation for empirical investigators to recommend that, in addition to research on personal-level behavioral strategies (e.g., *compartmentalization*) and individual-level attributes (e.g., *perseverance*), more studies should explore the merits of social and structural factors at the community level (e.g., *community-based health and social services*) to extend and expand much-needed HIV resilience resources [20,66,67].

*Compartmentalization* has been a behavioral strategy that many (including racial and ethnic minority) MSMLWH have come to rely on over the years to gain a degree of protection from the negative impacts of barriers to promoting HIV resilience such as racism and HIV stigma [68,69,70,71]. Although compartmentalization may not directly promote HIV resilience among racial and ethnic minority MSM, its protective effects prevent the decline of their resilience and provides them a reprieve from the sometimes unrelenting harmful effects of systemic factors such as racism and HIV stigma on their wellbeing. This reprieve further allows racial and ethnic minority MSM to sustain their perseverance to thrive even during the most challenging times after their HIV diagnosis. Research has shown that *perseverance* is an individual-level trait (similar to other protective factors like optimism and self-efficacy) that reduces risks of mental health problems among MSMLWH, and helps them deal with physical and psychosocial adversities related to living with HIV/AIDS [72,73]. 

The development and creation of community-level resources to support individual-level strategies and attributes for promoting resilience to HIV/AIDS among MSM would be a reasonable and promising approach, especially in the case of racial and ethnic minority MSM. Based on our participants’ responses, community-level resources, such as *community-based health and social services* (i.e., community health centers and ASOs), are critical to promoting their resilience to HIV/AIDS. Research has recognized the value of community health centers in ascertaining the early diagnosis of HIV and other STIs among MSM [74]. Research has also documented that community health centers have been vital to fostering regular screening of STIs and support for the continuity of HIV/AIDS care (e.g., diagnosis, treatment, follow-ups) for MSM [75]; greater accessibility to sexual health services (e.g., STI testing, provision of lubricants and condoms) and new knowledge [76]; and the consistent engagement of MSM in the healthcare delivery system prior to an HIV diagnosis and this engagement’s important connection to subsequent desired health outcomes [77]. The work of ASOs has led to increased opportunities for community involvement among MSM [78]; heightened awareness of concerns and issues relevant to the larger LGBTQ community [79]; and paths for developing social support networks and individual self-esteem [80]. Since the early 1980’s, ASOs have been the most accessible community resource for reliable information and updates on HIV/AIDS [81,82,83]. ASOs have also become dependable agencies for attaining information on essential services that MSMLWH require [82]; volunteering and stimulating activism to support greater funding for HIV/AIDS research [84]; and supporting the greater and meaningful involvement of PLWH (GIPA/MIPA) in the conceptualization and design of programs, services, research activities, and interventions that would positively impact their physical and mental health outcomes [85]. 

## 5. Limitations and Implications of the Study

In order to present a balanced discourse of our findings, it is important for us to acknowledge certain limitations of our study. First, we would like to emphasize the noteworthy influence of regional location and the Canadian context on our findings. Despite our best efforts to reach more participants from the rural areas of Ontario, the majority of our interviews were with participants from downtown Toronto (71%, *n* = 17) who had relatively easy access to the community-based health and social services they needed. This would explain why community-based health and social services were one of the most prominent themes related to facilitators to promoting resilience to HIV/AIDS. It would be reasonable to assume that this theme may not necessarily have been as prominent in our analysis if we had more participation from stakeholders residing in areas with less access to such services. The implication of this consideration is that there may be a need for community-based participatory researchers and other scholars to foster more advocacy and action for developing and creating additional community-based health and social services in more rural, suburban, and peri-urban locations to make these services more readily accessible to a greater number of middle-aged and older MSMLWH. In relation to this, looking into an even larger social context, as Canadians, our participants have had access to provincially and federally funded community-based health and social services for all, which may not be the case for many other middle-aged and older MSMLWH who may be residing in regional locations outside of Canada without access to universal healthcare or health services. Since they have had access to universal healthcare and social services, our participants may not have had to struggle as much with obtaining and paying for care and services they needed compared to other MSMLWH without access to universal healthcare and social services but with similar challenges and barriers to promoting HIV resilience as they did. 

Second, as a qualitative study that utilized a semi-structured interview guide for the purpose of acquiring a large data set, the interview questions we formulated with our community partners were designed to elicit responses that would expectedly develop numerous relevant themes. However, in order to remain focused in our analysis and presentation of our findings, it was necessary for us to choose to discuss only the most prominent themes we identified thematically. This would mean that from a pragmatic sense, some of the themes that we identified during our analysis of data would not necessarily have enough content or salience in the interviews to warrant discussion in this article. For example, although we acknowledge that themes such as ageism, xenophobia, homo/biphobia, specific mental and sexual health issues, and intersectionality were mentioned by some participants, there was not enough content in the interviews to generate a sufficient discussion about these themes based on the perspectives and lived experiences of those who participated in our study. This does not imply that these themes were not potential barriers to the promotion of resilience to HIV/AIDS among our participants based on their lived experiences. However, it does imply that these themes were not prominent enough to be considered as the major themes of our study during the analysis of our interview data. 

Finally, as a qualitative study, it is apparent that the findings from our interviews should be considered carefully and specifically to the context of our participants’ perspectives and lived experiences as middle-aged and older MSMLWH who have been residing in Ontario, Canada, in the last few decades. This implies that the perspectives and lived experiences of other MSMLWH in contexts and settings different from our participants’ may or may not be essentially reflected by our findings and analysis, depending on their race/ethnicity, age, regional location, access to services, and other pertinent factors. More empirical studies on resilience to HIV/AIDS among MSMLWH in different contexts and settings need to be conducted to explore the greater generalizability and applicability of our study findings. 

## 6. Conclusions

The findings and lessons learned from our study build on and extend existing knowledge on critical factors that promote resilience to HIV/AIDS, which have been examined and documented by prior empirical research. Our study explored and identified barriers and facilitators to promoting resilience to HIV/AIDS based specifically on the perspectives and lived experiences of participants who continue to be part of the population that is adversely affected by the clinical and social impacts of HIV/AIDS and syndemic factors related to their intersecting identities as HIV-positive, racial and ethnic minority, middle-aged and older MSM. The findings of our study not only underscored the merits of individual strategies and attributes such as compartmentalization and perseverance in the promotion of resilience to HIV/AIDS, but they also highlighted the importance of community-based health and social services such as community health centers and ASOs in fostering HIV resilience, particularly among racial and ethnic minority MSMLWH.

Although much of the research conducted on ASOs has focused on the mainstream and fairly established types of ASOs, as far as we could determine, there have been scant empirical investigations done on the intrinsic value of ethnoracial minority-serving ASOs to racial and ethnic minority MSMLWH and the promotion of their resilience to HIV/AIDS. While there have been a few ethnoracial minority-specific HIV support programs that have been launched in the last decade that show promise, further program evaluation and research efforts are still necessary to capitalize on the findings of our study, particularly on the specific merits to developing and establishing diverse ethnoracial minority-serving ASOs (and prospectively, community health centers), which are mostly found only in large metropolitan cities (i.e., Toronto, Los Angeles, New York). The purposeful establishment of ethnoracial minority-serving community health centers and ASOs to promote the HIV resilience of racial and ethnic minority MSMLWH could offset the stark differences in individual attributes and strategies among MSM and help address ongoing HIV/AIDS disparities affecting them. 

## Figures and Tables

**Table 1:** Participant sociodemographics (*n* = 24).

Race/Ethnicity	*n* (%)
African/Caribbean Black	8 (33)
Hispanic/Latino	4 (17)
Indigenous/Aboriginal	2 (8)
South/Southeast/East Asian	7 (29)
West Asian/Middle Eastern	3 (13)
Age Range	*n* (%)
40–44 years old	5 (21)
45–49 years old	7 (29)
50–54 years old	5 (21)
55–59 years old	2 (8)
60–64 years old	3 (13)
65–69 years old	0 (0)
≥70 years old	2 (8)
Identifies as	*n* (%)
gay	18 (75)
bisexual	3 (13)
MSM	1 (4)
two-spirit	2 (8)
Geographical Location	*n* (%)
Downtown Toronto	17 (71)
Greater Toronto Area	5 (21)
Southwestern Ontario	2 (8)

**Table 2:** Examples of interview guide questions.

Overview
Please tell us about yourself.
Please tell us how you believe you have remained healthy (since you were diagnosed with HIV)?
Social Relationships
What has your experience been with intimate relationships?
What has your experience been with relationships with partners, family, friends, informal caregivers, co-workers, etc.?
Meeting Men Who Have Sex with Men (MSM)
Where do you go to socialize with other MSM?
How do you meet people for sex?
Health, Social, and Other Services
Could you please tell me about your experiences accessing and navigating different health services?
Could you please tell me about your experiences accessing and navigating non-profit organizations, including AIDS services organizations?
Staying Healthy and Personal Health Practices
What kind of sex have you had or engaged in?
What are the different things in your life that have kept or are keeping you healthy?

**Table 3:** Themes and sub-themes.

Themes Related to Barriers to Promoting Resilience to HIV/AIDS
language proficiency
racism
pernicious norms in North American gay culture
hiv stigma
Themes Related to Facilitators to Promoting Resilience to HIV/AIDS
compartmentalization
perseverance
community-based health and social services

## Data Availability

The data presented in this study are available on request from the corresponding author. The data are not publicly available due to privacy and ethical reasons.
